# Field assessments in western Kenya link malaria vectors to environmentally disturbed habitats during the dry season

**DOI:** 10.1186/1471-2458-4-33

**Published:** 2004-08-05

**Authors:** John C Carlson, Brian D Byrd, Francois X Omlin

**Affiliations:** 1 Department of Tropical Medicine Tulane University, 1430 Tulane Avenue SL-17 New Orleans, LA 70112, USA; 2 International Centre of Insect Physiology and Ecology, PO Box 44, Kisii, Nyanza Province, Kenya

## Abstract

**Background:**

Numerous malaria epidemics have occurred in western Kenya, with increasing frequency over the past 20 years. A variety of hypotheses on the etiology of these epidemics have been put forth, with different implications for surveillance and control. We investigated the ecological and socioeconomic factors promoting highland malaria vectors in the dry season after the 2002 epidemic.

**Methods:**

Investigations were conducted in Kisii District during the dry season. Aquatic habitats in were surveyed for presence of malaria vectors. Brick-making pits were further investigated for co-associations of larval densities with emergent vegetation, habitat age, and predator diversity. Indoor spray catches were completed in houses near aquatic habitats. Participatory rural appraisals (PRAs) were conducted with 147 community members.

**Results:**

The most abundant habitat type containing *Anopheles *larvae was brick-making pits. Vegetation and habitat age were positively associated with predator diversity, and negatively associated with mosquito density. Indoor spray catches found that houses close to brick-making sites had malaria vectors, whereas those next to swamps did not. PRAs revealed that brick-making has grown rapidly in highland swamps due to a variety of socioeconomic pressures in the region.

**Conclusion:**

Brick-making, an important economic activity, also generates dry season habitats for malaria vectors in western Kenya. Specifically, functional brick making pits contain less that 50% as many predator taxa and greater than 50% more mosquito larvae when compared with nearby abandoned brick making pits. Further evaluations of these disturbed, man-made habitats in the wet season may provide information important for malaria surveillance and control.

## Background

The World Health Organization estimates that 300 to 500 million people are diagnosed with malaria annually, causing 1.1 to 2.7 million deaths. Approximately 1 million of these deaths are among children in sub-Saharan Africa, where 90% of all malaria cases occur [1]. During the 1950's and 1960's, a coordinated world-wide effort succeeded in eliminating malaria transmission within countries with temperate climates, and dramatically reduced malaria transmission in many other countries. Since the collapse of this campaign, malaria has since resurged, surpassing pre-campaign infection rates in many places, and entering previously unaffected locations [2]. The global resurgence of malaria has been attributed to a number of factors: drug-resistant parasites, insecticide-resistant vectors, population shifts, war-damaged infrastructures, altered meteorological conditions, and drastic ecological transformation [1,2].

The first recorded epidemic of malaria in the highlands of western Kenya occurred in 1918/19, with additional epidemics occurring periodically until 1950 [3]. These initial epidemics were associated with both population movements and progressive construction of roads and a railway through the highlands. With these human activities, new aquatic habitats were created, facilitating a gradual spread of parasites and vector mosquitoes into the highlands from the low-lying hyperendemic-disease areas [4]. Adoption of an extensive malaria control program [5] temporarily kept the highlands free from epidemics.

Since the 1980s, however, the incidence of highland malaria and frequency of epidemics have been increasing, with severe outbreaks in 1995, 1998/99, [6-9] and most recently in May through July, 2002. Fifteen districts are now considered epidemic-prone by the Kenyan government [10]. Climate changes have poorly predicted epidemics in the East African highlands [11-14]. Hay et al. [11] suggest that intrinsic population dynamics are more likely the cause of vector fluctuations. Investigators reviewing Kericho District tea plantation records [9,15,16] found no obvious change in average temperature or rainfall over the previous 20–30 years, stable tribal/ethnic composition in the study area, and a properly maintained health care system. These studies suggest a failure of medications was the most likely cause for the recurrence of malaria epidemics in the highlands. However, due to the retrospective approach, these studies were unable to investigate environmental/ecological changes in the highlands or evaluate the role of malaria vectors, as had been done for earlier epidemics [4].

Following the 2002 epidemic, this study was conducted to identify the ecological and socioeconomic variables affecting malaria vector densities and distributions. In this paper we connect specific man-made aquatic habitats present during the dry season with malaria vector larvae in western Kenya.

## Methods

### Study area

The highlands of western Kenya are an ecologically diverse and densely populated region situated east of Lake Victoria. Detailed investigations have been conducted in Mosocho Division, a sub-section of Kisii Central District with a predilection for epidemics and high rates of malaria (Ministry of Health Kisii, personal communication).

Kisii District covered 648 Km^2 ^and contained 491,786 people in 2001 (759 people/Km^2^) [17]. Mosocho Division covered 97 hectares and contained 105,309 people (1,086 people/Km^2^). Agriculture was the primary industry in the area, conducted on family plots which surrounded smaller industries, such as quarrying, and extended to the borders of wetlands.

### Survey of aquatic habitats in Mosocho Division

All sites with standing water within Mosocho Division were evaluated by standard dipping for presence of *Anopheles *mosquito larvae over the course of two weeks in the dry season (September 2002). Standing water was identified by canvassing Mosocho Division by vehicle and by foot, with the assistance of local inhabitants in the areas investigated. This technique especially relied on information from inhabitants when locating the more remote habitats, which may therefore be under-represented in the survey. Once identified, habitats were evaluated for presence of mosquito larvae using standard aquatic dippers. Dipping was performed around the perimeter of the habitat, with three dips performed at approximately one meter intervals. No effort was made to determine larval densities due to the patchiness of larval distribution within each habitat. Dipping was not performed more than one meter into the habitats, which may have led to an under representation of less abundant species.

### Cross-sectional survey of brick-making pits in Kisii District

**Figure 1:**
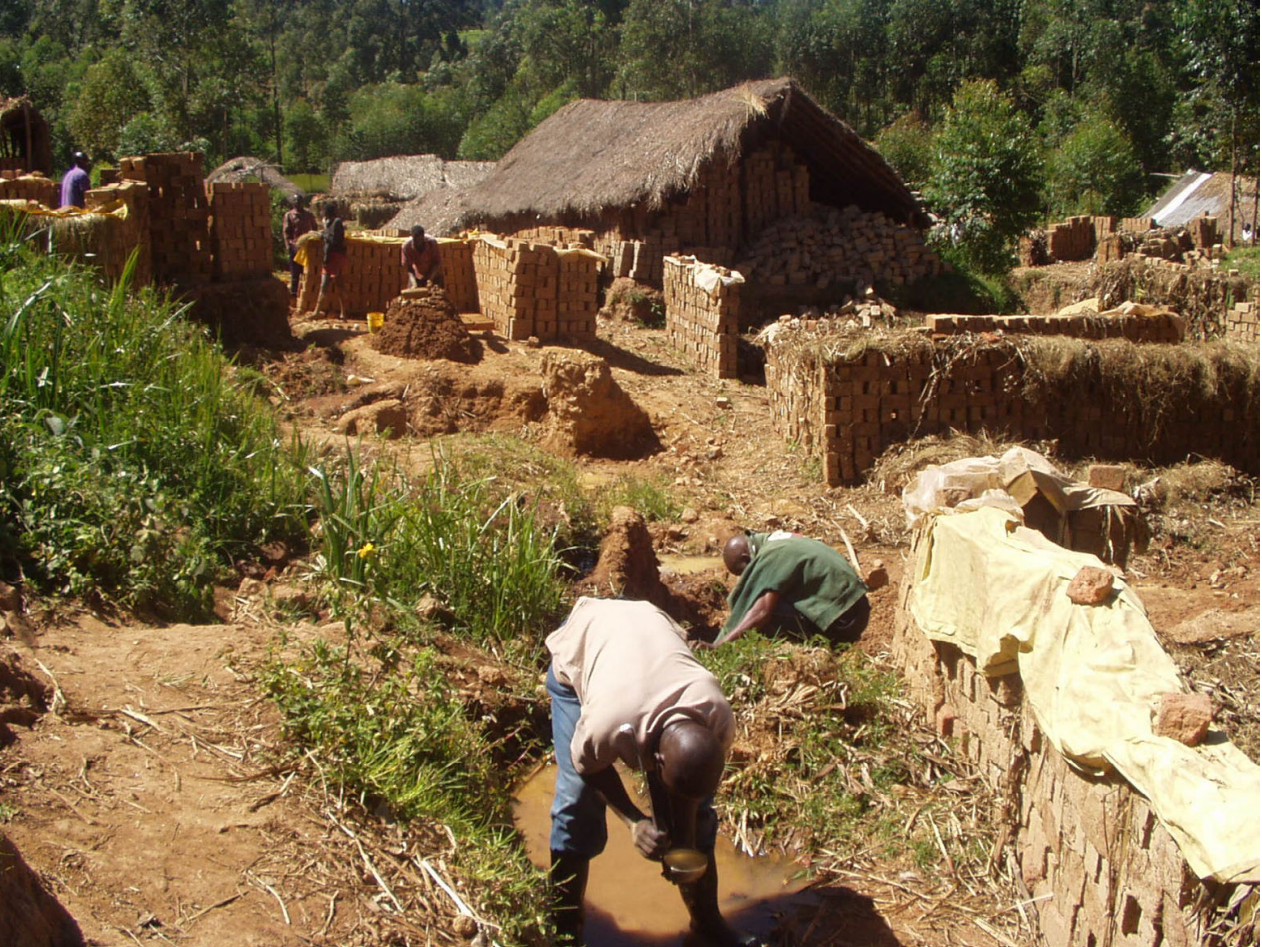
**Brick-making site in the Kenyan highlands. **Assessments of the shallow pools dug by brick-makers is taking place in the foreground as brick makers continue to excavate soil, combine the soil with water, mould the resulting mud into bricks, and then left to dry before firing.

At each of five brick-making sites in Kisii District, three functional brick-making pits and three abandoned pits were evaluated for pit size, larval densities, percent emergent vegetation and predator taxa present in the habitat. (See Figure 1.) Functional pits were chosen by asking brick makers to identify sites in which bricks were currently being produced that had been in use for at least two weeks. Abandoned pits were chosen near functional pits that had not been used for brick-making during the previous four months according to brick makers. Surface area of the rectangular pits was estimated by multiplying the measurements of the habitat at maximum and minimum lengths. Larval densities were obtained by standard dipping method, averaging the yields of five dips per pit. Percent emergent vegetation was estimated visually. An estimation of predator diversity was obtained by adding the number of Vertebrata orders, Odonata suborders, Hemiptera families, and adult Dytiscidae (Coleoptera) size-categories collected from the pits with 20 cm diameter fine-mesh sieves capable of collecting organisms down to 1 mm in size[18]. Predators were surveyed after obtaining larval dips, with one minute spent in each pit.

### Spatial data

To assess the spatial relationship between aquatic habitats and the presence of malaria vectors inside houses, a series of indoor spray catches (described by [19]) was performed on 5 September 2002. Nine houses were chosen along a transect from a valley used for brick-making up to a maximum elevation (1920 meters), and then down into the next valley, containing swamps. Houses were chosen for possession of a thatched roof, not having had a fire inside since the previous evening, and permission of the owner.

### Socioeconomic survey

Forty nine Chiefs and ninety eight brick makers were interviewed at four locations within Kisii and Gucha Districts to determine the social and economic factors promoting brick-making in the highlands, as well as the historic development of the industry. Standard Participatory Rural Appraisal methodology was used to obtain qualitative data [20].

### Statistical analysis

The means of the variables in the cross-sectional survey were compared for independence between the 15 functional and 15 abandoned brick-making pits (SPSS 11.0 t-test for independence).

GPS coordinates taken for correlating presence of *Anopheles *in a house with distance to brick-making pits and swamp were converted into decimal degree coordinates and integrated as map layers within a Geographical Information System (ArcView 8.2). The distances from each house to the nearest brick making pit and swamp boundary were generated using the distance tool within the ArcView software. Spearman's correlation coefficient (SPSS 11.0) was calculated to correlate presence of *Anopheles *in a house with distance to swamp, distance to brick-making pits, and elevation.

## Results

### Survey of aquatic habitats in Mosocho Division

A dry-season survey of aquatic habitats in Mosocho Division was conducted to identify potentially important larval habitats of *Anopheles *mosquitoes. (See Table 1.) A total of 53 standing aquatic habitats were assessed, with 16 (30.2%) containing *Anopheles *larvae. 37.5% of *Anopheles*-positive habitats were functional brick-making sites containing only *An. gambiae s.l. *larvae, and 18.8% were abandoned brick-making habitats containing only *An. gambiae s.l. *larvae (combined for 56.3%). Of the four quarries positive for *Anopheles *larvae, three were functional (created in the previous year) and one was abandoned (unused > two years), both with *An. gambiae s.l *and *An. funestus*. A portion of a drainage canal dug near a brick-making sites constituted an additional *Anopheles*-positive habitat, containing *An. gambiae s.l*. larvae. Of natural habitats sampled, only two contained malaria vectors. One swamp had *An. funestus *larvae and one tree hole in an ornamental Flamboyant tree (*Delonix regia*) had *An. gambiae s.l. *larvae; 87.5% of *Anopheles*-positive habitats were of direct human origin.

**Table 1:** Dry season survey of aquatic habitats

**Habitat**	**Number of assessed habitats**	**Number of habitats with *Anopheles *larvae**	**% of habitats with *Anopheles *larvae**	**% of total *Anopheles *larval habitats**
**BMS (F)**	6	6	100	37.5
**Quarry (F)**	4	3	75	18.8
**BMS (A)**	14	3	21.4	18.8
**Quarry (A)**	10	1	10	6.3
**Tree hole**	5	1	20	6.3
**Swamp**	6	1	16.7	6.3
**Drainage**	7	1	14.3	6.3
**Fish pond**	1	0	0	0
**Stream pool**	2	0	0	0
**Total**	53	16	30.2	100.3

BMS (F): Functional Brick-making site BMS (A): Abandoned Brick-making site

### Cross-sectional survey of brick-making pits in Kisii District

Mean mosquito larvae densities were higher in functional than abandoned pits for both *An. gambiae s.l*. (2.87/dip in functional, 0.91/dip abandoned, p = 0.002) and *Culex *spp. (3.77/dip in functional; 1.32/dip in abandoned, p = 0.025). No *An. funestus *larvae were found in this study. This corresponded with an increase in predator biodiversity found in the abandoned sites (9.07 taxa in abandoned; 5.13 taxa in functional, p= 0.001) and increased percent emergent vegetation (0.27% in functional; 94.27% in abandoned, p < 0.001). (See Table 2.) Functional brick-making pits were an average of 7.8 square meters (standard deviation= 6.38; range = 1.65 to 22.5 square meters).

**Table 2:** Ecological survey of brick-making pits

	**Use**	**N**	**Mean**	**Std. Deviation**	**t-statistic**	**p**
**Percent vegetation**	Functional	15	0.27	0.46	-47.116	<0.001
	Abandoned	15	94.27	7.71		
**Habitat age (months)**	Functional	15	0.62	0.17	-5.734	<0.001
	Abandoned	15	24.20	15.92		
**Predator biodiversity**	Functional	15	5.13	2.36	-3.586	0.001
	Abandoned	15	9.07	3.53		
**Average number of Anopheles/ dip (5 dips per pit)**	Functional	15	2.87	2.04	3.402	0.002
	Abandoned	15	0.91	0.90		
**Average number of Culex/dip**	Functional	15	3.77	3.60	2.435	0.025
	Abandoned	15	1.32	1.51		

### Spatial data

Spray catches in nine houses during the dry season resulted in one *Anopheles *mosquito in five different houses. Distance from brick-making sites was negatively correlated with presence of *Anopheles *in houses (p = 0.002, r = -0.868), while distance from swamp was positively correlated with presence of Anopheles mosquitoes (p = 0.018, r = 0.758). Elevation was not correlated with presence of *Anopheles *in a house (p = 0.829, r = -0.085).

### Brick-making and socioeconomic survey

According to brick-makers, making bricks is predominantly a dry-season activity due to the damage caused by heavy rains to drying bricks. Although variation in technique exists, universally used stages in the brick-making process are: Excavation, Fermentation, Moulding, Drying, and Kilning. During the Moulding stage, water is brought into the excavated clay pits and mixed with soil. During this stage, which lasts from several days to one month, water is continually supplied to the pit through irrigation systems, ground water, or is brought by bucket from a nearby water source. Once abandoned during the Kilning stage and afterwards, the pit accumulates rain water and ground water, which can be subsequently used as a water source for newly excavated pits. Over years, abandoned brick-making pits degrade until they are continuous with the surrounding swamp.

Interviews with Chiefs and brick makers revealed different scales of brick-making operations, from individuals working on their own land to large-scale industries (Figure 1) where wetland plots are rented to brick-makers who employ a large number of casual laborers for the mass production of bricks. Brick-making is an increasingly popular means of obtaining income, spreading to new communities when skilled brick makers are hired to work new land for short-term projects. While brick-making has been passed through multiple generations, the large-scale industries have developed steadily over the previous 20 years.

A primary socioeconomic factor described by brick-makers that promotes brick-making is the desire to make use of agriculturally unfavorable wetlands as human population densities increase. Nearly all brick makers work in wetlands, leading to progressive deforestation of the highland swamps. Money from brick-making was used principally for paying school fees and malaria medication. According to brick-makers, brick houses are considered to be more prestigious than traditional houses, are associated with lower long-term costs than traditional mud-thatched houses, and are less permeable to mosquitoes.

## Discussion

Our study shows that man-made larval habitats were the predominant (87.5%) source of malaria vectors in Mosocho Division during the dry season. In particular, the continuously disturbed, functional brick-making pits contained high densities of malaria-vectoring mosquitoes. PRAs revealed that small brick-making groups have developed into large-scale industries over the past two decades, and brick-making is now dispersed throughout the highlands in unfarmable wetlands. Because brick-making occurs predominantly in the dry season, it may aid in maintaining vector populations year-round.

Additionally, houses closest to brick-making pits had malaria vectors present within them. While the number of mosquitoes captured during a single transect of spray-catches was only one per house, this represents a real, if low, possibility of malaria transmission during the dry season at these locations. Thus brick-making areas may function as refugia for malaria parasites and their vectors over the dry season, facilitating spread of malaria when habitats become more plentiful in the wet season.

In this study we found emergent vegetation to be negatively associated with presence of malaria vectors in man-made habitats. This association may be a result of an association of both emergent vegetation and predator diversity with habitat age. In continually disturbed habitats (such as functional brick-making pits), the habitats are kept at a low stage of biological succession, possessing fewer species of both plants and animals. There may also be a direct effect of vegetation on the trophic dynamics in ground pools. In structurally simple habitats, intraguild predation has been shown to suppress the diversity of important predators [21-23]. Whether due to co-associated variables, direct effects, or some combination, there was a sizable 57% increase in predator taxa found in abandoned, vegetated pits and a >50% reduction of *Anopheles *larval densities. More research into the ecology of small aquatic pools will help clarify the interrelationships of these variables.

In ground pools, predators are typically thought to regulate the density of mosquito larvae [24,25]. Predators may exert an effect by consuming larvae or through deterring oviposition into an otherwise suitable habitats[26]. During the dry season, disturbed, man-made habitats (such as functional brick-making pits and quarries) provide a developmental habitat in which *Anopheles *larvae escape the high degree of predation found in the natural environment. These habitats should be specifically targeted during larval control programs. With substantial socioeconomic motivation for brick-making in the highlands, traditional source reduction (eliminating standing water) is unsustainable, and larvicides should be employed. Further research is needed on the use of these habitats by mosquito larvae during the wet season.

The negative correlation between predator diversity and mosquito density suggests that pesticide application may exacerbate epidemics by decreasing predation pressure if used in natural habitats. Pesticide applications can be selectively harmful to larger predators such as dragonflies and large dytiscid beetles which take months to years to develop[27]. In contrast, larvicides have a smaller impact on rapidly developing insects such as mosquitoes, which can reach maturity in a week. Rapid recolonization of predator-free habitats by mosquitoes would lead to vector resurgence [8,28]. Thus, targeting larvicide application to disturbed aquatic habitats should lead to a better mosquito control than treating all available habitats.

The social and economic benefits accompanying disease reduction through vector control was demonstrated in the Zambian copperbelt [29]. As the population density of the highlands of western Kenya grows, the social and economic costs of malaria are likely to grow as well, unless vector-centered interventions (including environmental management, larvicide application, and vector surveillance systems) are used to confront the disease.

## Competing interests

None declared

## List of abbreviations

PRA: Participatory Rural Appraisal

Km: Kilometer

BMS: Brick making site

NGO: Non governmental organization

## Authors' contributions

JCC designed and conducted evaluations of larval habitats and houses, performed statistical analysis, and wrote the initial draft of the manuscript. BDB performed GIS analysis. FXO identified BMS as sources of vector mosquitoes, designed and conducted PRAs, and aided in the production of the manuscript's final draft.

## Pre-publication history

The pre-publication history for this paper can be accessed here:


